# Immune cell networking in solid tumors: focus on macrophages and neutrophils

**DOI:** 10.3389/fimmu.2024.1341390

**Published:** 2024-02-14

**Authors:** Irene Di Ceglie, Silvia Carnevale, Anna Rigatelli, Giovanna Grieco, Piera Molisso, Sebastien Jaillon

**Affiliations:** ^1^ IRCCS Humanitas Research Hospital, Milan, Italy; ^2^ Department of Biomedical Sciences, Humanitas University, Milan, Italy

**Keywords:** tumor microenvironment, anti-tumor immunity, tumor-associated macrophages, tumor-associated neutrophils, immune cell network

## Abstract

The tumor microenvironment is composed of tumor cells, stromal cells and leukocytes, including innate and adaptive immune cells, and represents an ecological niche that regulates tumor development and progression. In general, inflammatory cells are considered to contribute to tumor progression through various mechanisms, including the formation of an immunosuppressive microenvironment. Macrophages and neutrophils are important components of the tumor microenvironment and can act as a double-edged sword, promoting or inhibiting the development of the tumor. Targeting of the immune system is emerging as an important therapeutic strategy for cancer patients. However, the efficacy of the various immunotherapies available is still limited. Given the crucial importance of the crosstalk between macrophages and neutrophils and other immune cells in the formation of the anti-tumor immune response, targeting these interactions may represent a promising therapeutic approach against cancer. Here we will review the current knowledge of the role played by macrophages and neutrophils in cancer, focusing on their interaction with other immune cells.

## Introduction

To develop and grow, tumor cells require constant support from cells of the surrounding environment (1–3). Immune cells are key players in this scene where they actively collaborate to either promote or inhibit tumor growth (4, 5). Through the production of numerous immunomodulatory molecules (e.g. cytokines, chemokines and growth factors), tumor cells can modulate the phenotype of immune cells and the positive or negative influence that they exert on the tumor microenvironment (TME) (6, 7). Additionally, tumor-infiltrated immune cells are engaged in a number of mutual interactions which further potentiate or reduce their pro-tumor or anti-tumor activities (8–10). Tumor-associated macrophages (TAMs) have long been identified as central players of this complex intercellular network (11–13). More recently, neutrophils, traditionally considered only as short-lived front-line fighters against pathogens, have received increasing attention due to their important role in regulating tumor development (14–17).

Being highly plastic and consequently heterogeneous, TAMs and tumor associated neutrophils (TANs) adapt their phenotype and activation state to the surrounding environment (11–21). TAMs and TANs have the capacity to act directly on tumor cells to promote or inhibit their proliferation, and to modulate the anti-tumor or pro-tumor activities of other immune cells (11, 14, 22). Targeting the immune system is now a reality and is emerging as an important therapeutic approach for the treatment of cancer (23, 24). However, a significant percentage of patients do not respond to current available treatments (25). Given the crucial importance of the intercellular crosstalk between immune cells within the TME, novel therapeutic approaches targeting these interactions might be beneficial. In this review, after a short overview concerning TAMs and TANs, we will focus on their complex intercellular interactions with other immune cells, with a particular emphasis on how these interactions can influence tumor development and progression.

## Macrophages in the tumor microenvironment

Macrophages are large phagocytic cells of the innate immune system and are found in tissues where they play numerous roles, including defense against invading pathogens and maintenance of tissue homeostasis (19). Macrophages have long been identified as tumor-infiltrating cells and as one of the key regulators of the immune response within the TME (11–13). In a majority of human cancers, a high level of macrophage infiltration has been associated with a poor prognosis, including in gastric cancer, urogenital and head neck cancers, pancreatic ductal adenocarcinoma and breast carcinoma with some notable exceptions such as colorectal cancer and ovarian cancer (26–33).

The majority of TAMs derive from circulating bone marrow (BM)-derived monocytes that migrate into the tumor bed mainly under the influence of chemokines (e.g. C-C motif ligand 2 (CCL2)), cytokine colony stimulating factor 1 (CSF-1), or complement components (26, 34–39). In addition, numerous studies have shown that another important source of TAMs was represented by tissue-resident macrophages (TRMs), which derive from embryonic precursors and are maintained locally (40–45). Interestingly, a difference in origin can influence the function of TAMs within the TME (46, 47).

In addition to their heterogenous origin, TAMs have a high degree of plasticity and constantly adapt in response to the microenvironment further increasing their heterogeneity within the TME (11, 19, 48, 49). Historically, TAMs have been classified into two major polarization states, generally referred to as M1 (or classically activated) and M2 (or alternatively activated) (50–52). M1 polarization can be induced by bacterial products and interferon-γ (IFN-γ) and has been associated with tissue damage and anti-tumor activities (50, 51). In contrast, M2 polarization, induced by cytokines of the type 2 immune response, such as interleukin (IL)-13 and IL-4, has been associated with tissue repair and tumor-promotion (50, 51, 53). Although this dichotomous classification is still commonly used, it is nowadays fully recognized that it is too reductive and does not reflect the extraordinary complexity and heterogeneity of the different phenotypes and activation states of TAMs (13, 21, 52). Novel technologies, including single-cell RNA sequencing and spatial transcriptomic analyses, have led to a better understanding of macrophage complexity and heterogeneity in different tumor contexts. In addition, these technologies have facilitated insight into the localization of macrophages and their interactions with other cells within the TME (54–61).

## Macrophages and immune cells cross-talk in the TME

### Macrophages and lymphoid cells

The existence of a crosstalk between macrophages and lymphoid cells within the TME has been clearly defined and extensively studied. In various human cancers, TAMs were found in close proximity to different lymphoid cells, including natural killer (NK) cells and T cells (62–64). The final effect of the interaction between macrophages and lymphoid cells can differ depending on the type and the stage of tumors, the presence of immunomodulatory molecules and the location of TAMs within the TME.

A large body of evidence has shown that TAMs can inhibit the anti-tumor activity of lymphoid cells, thereby promoting cancer progression. As a matter of fact, the presence of monocyte/macrophage infiltration was found to be inversely correlated with the presence and activation of NK cells in different human cancers including lung cancer, gastric cancer and hepatocellular carcinoma (HCC) (64–66). In contrast, monocyte/macrophage infiltration was found positively correlated with the presence of regulatory CD4^+^ T lymphocytes (T-reg) in prostate cancer and colorectal cancer (67, 68). Consistently, CSF-1– CSF-1 receptor (CSF-1R) axis blockade or macrophage recruitment blockade via C-C chemokine receptor type 2 (CCR2)-inhibition could restore immune response against tumor in cancer models (69, 70).

**Figure 1:**
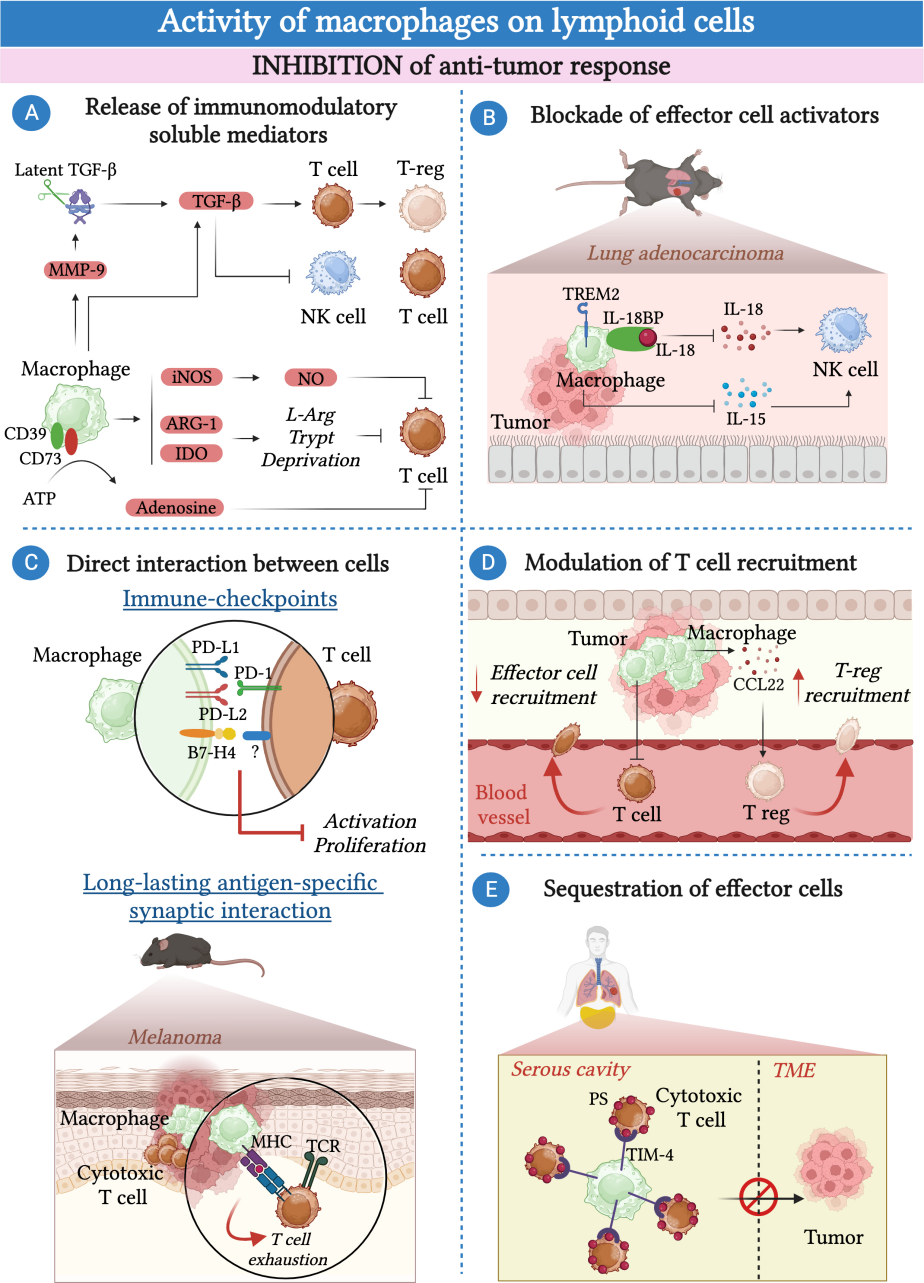
Macrophages inhibit the anti-tumor response of lymphoid cells. **(A)** Macrophages secrete a plethora of soluble molecules and immunomodulatory enzymes that inhibit effector cell functions including tumor growth factor β (TGF-β), inducible nitric oxide synthase (iNOS), indoleamine-2,3-dioxygenase (IDO) and arginase-1 (Arg-1). In addition, they produce metalloproteinase 9 (MMP-9) that induces the activation of the latent TGF-β present in the extracellular matrix (ECM). TAMs express CD39 and CD73, which convert the pro-inflammatory ATP present in the TME into the immunosuppressive adenosine. **(B)** In the context of lung carcinoma, triggering receptor expressed on myeloid cells 2 (TREM2)-expressing macrophages suppress the cytokine-mediated activation of NK-cells via the production of IL-18-binding protein (IL-18BP) and by limiting IL-15 production by dendritic cells. **(C)** Macrophages can inhibit the activation of effector cells in a contact-dependent manner. They express immune checkpoints such as programmed cell death ligand 1 (PD-L1), PD-L2 and B7-H4 that, by binding to their ligands, impair the activity of T cells and NK cells. In a mouse model of melanoma, the formation of an antigen-specific interaction between macrophages and T cells leads to T cell exhaustion. **(D)** Macrophages can inhibit the recruitment of effector T cells into the TME whereas they favor the recruitment of T-reg lymphocytes via the secretion of CCL22. **(E)** In pleural and peritoneal body cavities, macrophages expressing T-cell immunoglobulin and mucin domain containing 4 (TIM-4) can sequester CD8^+^ cytotoxic T cells away from the tumor by binding to phosphatidylserine (PS) expressed on their surface and inhibiting their proliferation.

The mechanisms by which TAMs can inhibit the anti-tumor activities of lymphoid cells are numerous ranging from the release of soluble inhibitory mediators and cell-cell contact to the modulation of their recruitment or their exclusion from the tumor bed (**Figure 1**). The release of tumor growth factor-β (TGF-β) by TAMs can directly inhibits T cell and NK cell effector functions (64, 71–74). In addition, macrophage-derived TGF-β can promote the generation of T-reg lymphocytes via the induction of SMAD3-mediated FOXP3 expression and the upregulation of PD-1 (68, 75). TGF-β availability within the TME can be further amplified by metalloproteinase 9 (MMP-9) secreted by macrophages, which can cleave and activate latent TGF-β present in the extracellular matrix (ECM) (76).

TAMs can release the immunoregulatory enzymes Arginase-1 (Arg-1) and indoleamine 2,3-dioxygenase (IDO) that, by catalyzing the degradation of L-arginine (L-arg) and L-tryptophan respectively, deprive T cells of essential nutrients, leading to their functional impairment (77–79). A third important immunomodulatory enzyme produced by macrophages is the inducible nitric oxide synthase (iNOS), which catalyzes the production of nitric oxide (NO). In turn, NO has a direct inhibitory effect on T cell proliferation, as well as an indirect effect due to the secondary production of peroxynitrites able to impair the interaction of the major histocompatibility complex (MHC) with the T cell receptor (TCR) via nitration of tyrosines in the TCR-CD8 complex (80–82).

TAMs can exert their pro-tumor activity by blocking the activity of soluble factors that normally contribute to the activation of lymphoid cells. For instance, TAMs can express CD39 and CD73, which are essential in converting the pro-inflammatory adenosine triphosphate (ATP) present in the TME into immunosuppressive adenosine (83). In glioblastoma, kynurenine produced by cancer cells upregulates the expression of hydrocarbon receptor (AHR) in TAMs. In turn, AHR favors the expression of the ectonucleotidase CD39, which, in collaboration with CD73, leads to the dysfunction of CD8^+^ T cells via the production of adenosine (84, 85).

In a murine model of lung adenocarcinoma, efferocytosis of apoptotic cancer cell by macrophages induced a pro-tumorigenic program controlled by triggering receptor expressed on myeloid cells 2 (TREM2). In particular, TREM2^+^ macrophages can prevent the recruitment and activation of NK cells by blocking the activities of IL-18 and IL-15 through the production of IL-18 binding protein and by inhibiting the production of IL-15 by tumor-infiltrating dendritic cells (65).

The mechanism by which macrophages can dampen the anti-tumor response goes beyond the secretion of molecules or the interference with soluble mediators and often requires direct cell-cell contact.

TAMs have been found to express high levels of immune checkpoint molecules, including programmed cell death ligand 1 (PD-L1), PD-L2, V-domain Ig suppressor of T cell activation (VISTA) and B7-H4 (11, 86–93). The interaction of these immune checkpoints with their ligands expressed on T cells results in the suppression of the adaptive T cell immune response (94). Consistently, immune checkpoint blockade can restore T cell-mediated anti-tumor immune response (89, 91). In addition to T cells, PD-1, the ligand of PD-L1, can be expressed by a subset of fully mature NK cells, suggesting that NK cells can also serve as a target of PD-L1-mediated inhibition by macrophages (95).

In addition to the expression of immune checkpoints, other mechanisms involving cell-cell contact between macrophages and T cells have been identified and may lead to T cell exhaustion. For instance, TAMs and CD8^+^ T cells can be engaged in a long-lasting antigen-specific synaptic interaction. This interaction causes only weak stimulation of the TCR, which is insufficient to activate T cells and instead leads to their exhaustion (96).

Furthermore, different lines of evidence suggested the involvement of TAMs in modulating the recruitment of lymphoid cells within the TME. In cervical and breast cancer models, CSF-1R-blockade has been shown to enhance CD8^+^ T cell infiltration (97). Consistently, LIF-mediated epigenetic silencing of CXCL9, one of the most important chemokines for CD8^+^ T cells, in macrophages resulted in decreased infiltration of CD8^+^ T cells into the tumor (98, 99). While they can reduce the recruitment of effector CD8^+^ T cells in the TME, macrophages have been suggested to facilitate the recruitment of T-reg lymphocytes via the secretion of CCL22 in human ovarian cancer (100). In a mouse model of melanoma, intra-tumoral administration of an anti-CCL22 antibody reduced the recruitment of T-reg lymphocytes and inhibited tumor growth (101).

Macrophages have been implicated in the sequestration of effector T cells away from the tumor bed. Recently, a study showed that serous cavity-resident macrophages expressed high levels of T-cell immunoglobulin and mucin domain containing 4 (TIM-4), a receptor for phosphatidylserine (PS). The interaction between TIM-4 and PS, highly expressed on cytotoxic CD8^+^ T cells, resulted in the sequestration of CD8^+^ T cells away from the tumor and inhibited their proliferation (102). Similarly, in samples of human non-small cell lung carcinoma (NSLC),TAMs reduced the motility of the CD8^+^ T cells present in the stroma surrounding the tumor, limiting their entry into the tumor bed (62).

**Figure 2:**
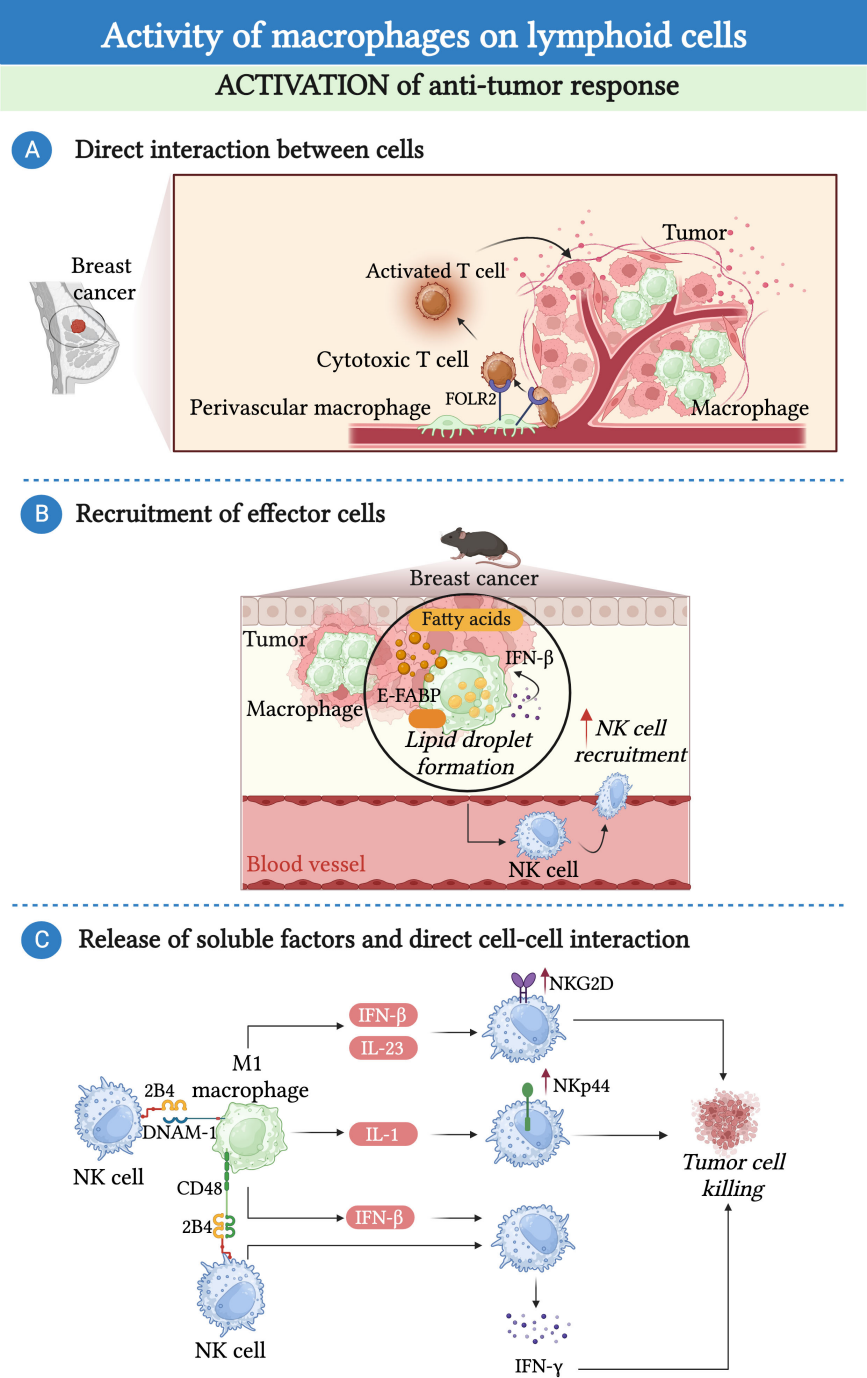
Macrophages promote the anti-tumor response of lymphoid cells. **(A)** In the context of breast cancer, a population of folate receptor 2 (FOLR2)-expressing macrophages endowed with anti-tumor properties can establish a prolonged interaction with cytotoxic T cells facilitating their recruitment and activation. **(B)** In a mouse model of mammary tumor, accumulation of lipid droplets in epidermal fatty acid binding proteins (E-FABP)-expressing TAMs induces the production of interferon-β (IFN-β), leading to the recruitment and activation of NK-cells. **(C)** Macrophages have the potential to support the anti-tumor activity of NK cells. M1-macrophages induce an IL-23 and IFN-β-dependent upregulation of NKG2D expression, an IL-1β-dependent upregulation of NKp44 expression, and sustained the production of IFN-γ by NK cells via the release of IFN-β and the interaction of CD48 and DNAX accessory molecule-1 (DNAM-1) with 2B4.

Although in a large number of studies, TAMs appeared to reduce the anti-tumor activity of lymphoid cells, some works have suggested the existence of macrophages with an opposite function in different tumors (**Figure 2**). Recently, a study identified a discrete population of human tissue-resident FOL2R^+^ macrophages present in healthy mammary gland and breast cancer primary tumors endowed with an anti-tumor function. This population of macrophages was localized in the perivascular areas of the tumor stroma where they interacted with CD8^+^ T cells and promoted their activation (103). Of note, the presence of FOLR2^+^ macrophages has been associated with increased survival in breast cancer patients (103). In addition to T cells, macrophages have the potential to support the anti-tumor activity of NK cells either via the release of soluble molecules or via direct contact. For instance, in a model of mammary tumor, TAMs expressing the lipid transporter epidermal fatty acid binding proteins-(E-FABP) have been described to have an anti-tumor activity through the activation of NK cells. Specifically, the expression of E-FABP promoted the formation of lipid droplets in TAMs, leading to increased IFN-β production, which, in turn, favored the recruitment of effector cells, particularly NK cells (104). Remarkably, the same study showed that E-FABP was highly expressed in TAMs from women with early-stage disease, and that this expression decreased with disease progression (104). In *in vitro* co-culture experiments, M1-macrophages induced an IL-23 and IFN-β-dependent upregulation of natural killer group 2D (NKG2D) expression, an IL-1β-dependent upregulation of NKp44 expression, and sustained the production of IFN-γ by NK cells via the release of IFN-β and the engagement of the 2B4-CD48 pathway (105). Similarly, M0 and M2 macrophages reprogrammed to M1 via *in vitro* LPS stimulation can promote the cytotoxic activity of NK cells via a contact-dependent mechanism and drive the production of IFN-γ by NK cells via the interaction between DNAX accessory molecule-1 (DNAM-1) and 2B4 and the production of IL-18 (73, 106).

In addition to T cells and NK cells, some evidence suggested that macrophages could promote B cell proliferation via the release of B cell-activating factor (BAFF) or IL-6 (8, 107, 108). However, the relevance of this interaction within the TME remains to be clarified.

The interaction between macrophages and lymphoid cells within the TME is mutual and the phenotype and function of macrophages can also be affected by lymphoid cells (12, 109). For instance, the IFN-γ produced by T-helper (Th)-1 cells, NK cells and cytotoxic CD8^+^ T cells increases the presentation of antigens by macrophages, the production of pro-inflammatory cytokines and the cytotoxic activity of macrophages against tumor cells (48, 109, 110). In contrast, lymphoid cells found in the TME can also favor a pro-tumor phenotype of macrophages. For instance, CD4^+^ Th-2 cells, innate lymphoid cells (ILCs)-2- and T-reg lymphocytes that produce IL-4, IL-13 and IL-10 can sustain the formation of M2-like macrophages with a pro-tumor phenotype (111–115). Interestingly, it has been shown that mouse T-reg lymphocytes can indirectly promote the survival of M2-like pro-tumor macrophages. Indeed, by limiting the production of IFN-γ by CD8^+^ T cells, T- reg lymphocytes prevent the inhibition of sterol regulatory element binding protein 1 (SREBP1)-mediated fatty acid synthesis, which is crucial for the survival of M2-like macrophages (116).

Additionally, NK cells have been shown to be able to kill macrophages. Of note, this macrophage killing activity was found to be especially efficient toward M0 and M2 macrophage subtypes whereas M1 macrophages were more resistant to lysis due to the higher expression of human leukocytes antigen (HLA) class I molecules (106). Similarly, invariant natural killer T cells (iNKT) have been shown to exert anti-tumor activity by killing macrophages in a CD1d-dependent mechanism (117).

Finally, some studies have highlighted an effect of B cells on macrophages in tumor. In a mouse model of melanoma, adoptive transfer of a subtype of B cells can induce M2-like polarization of TAMs (118).

### Macrophages and myeloid cells

While the interaction between macrophages and lymphoid cells has been the subject of numerous studies, their interaction with other myeloid cells has received less attention. Among myeloid cells, dendritic cells are key players in the orchestration of both innate and adaptive immune responses in cancer (119).TAMs produce high levels of vascular endothelial growth factor (VEGF), IL-10, IL-6 and TGF-β which are described to inhibit the activity of dendritic cells (11, 120–124). Besides dendritic cells, macrophages can interact with neutrophils (14). The interaction between these two important myeloid subtypes within the TME will be further discussed in this review.

## Neutrophils in the tumor microenvironment

Neutrophils are the most abundant circulating leukocytes in humans. They have long been considered as simple first-line fighters against invading pathogens, but are now recognized as central players in the regulation of tumor development and progression (14, 16). TANs have been found in the TME of several human cancers, including renal cell carcinoma, hepatocellular carcinoma, lung cancer, melanoma, head and neck cancer, glioma, colorectal cancer, sarcomas, pancreatic cancer, breast cancer, gastric cancer, urothelial carcinoma and ovarian cancer (125–136). However, their role is still controversial and may depend on a number of factors, including the different types of cancer, the stage of development and the presence of other cells (14, 17, 137). While in a large number of studies, a high level of TANs has been associated with a poor prognosis for patients, in others, including colorectal cancer and undifferentiated pleomorphic sarcoma (UPS), it has been associated with a better outcome (14, 125, 130, 138–143).

Mature mouse and human neutrophils are constantly released from the BM where they differentiate and mature from progenitors in response to growth factors, in particular granulocyte-colony stimulating factor (G-CSF) and granulocyte macrophage-colony stimulating factor (GM-CSF) (16, 144–149). The process of neutrophil mobilization from the BM has been extensively investigated in mice and is highly dependent on the regulation of the expression of genes coding for CXCR4 and CXCR2 (150). Upon maturation, BM-neutrophils downregulate CXCR4 expression, which is the receptor for CXCL12 produced by BM stromal cells, and increase the expression of CXCR2. The expression of CXCR2 and the presence CXCL2, which is the ligand for CXCR2, in the circulation trigger the release of neutrophils into the peripheral blood (150, 151). The observation of alterations in neutrophil biology among patients with genetic mutations in CXCR4 and CXCR2 implies the significance of these molecules also in human (152, 153). Stress conditions, including cancer, trigger an “emergency granulopoiesis” program during which the process of neutrophil maturation and BM egress is altered, resulting in the release of immature neutrophils into the circulation (154).

Circulating neutrophils express high levels of CXCR1 and CXCR2, which play a major role in their recruitment into the TME (14, 16, 155). Numerous studies have shown the involvement of CXCR1 and CXCR2 ligands, including CXCL1, CXCL2, CXCL5, CXCL6 and CXCL8 (only for humans) in the recruitment of neutrophils into the TME (14, 156–162). Additionally, other inflammatory mediators, including the cytokines TNF-α, IL-17 and IL-1β have been implicated in the recruitment of neutrophils into the TME (14, 163, 164).

While neutrophils were traditionally considered as short-lived effector cells with limited plasticity, a large body of evidence challenged this view and recognized their considerable plasticity and heterogeneity (17, 18, 20). Based on their phenotype and function and mirroring the M1/M2 paradigm, TANs have been classified into anti-tumor (N1) and pro-tumor (N2) neutrophils (14, 165, 166). As mentioned above for macrophages, new studies based on state-of-the-art methodology, including single cell RNA sequencing, mass cytometry by time-of-flight (CyTOF), multiplex immunofluorescence and spatial transcriptomic have revealed a high degree of heterogeneity in TANs (14, 16, 167–171).

Pro-tumor neutrophils can directly promote tumor development by inducing tissue damage and genetic instability through the production of radical oxygen species (ROS) and miRNA, and by stimulating tumor growth through the secretion of cytokines and growth factors (172–177). Additionally, pro-tumor neutrophils can facilitate the formation of tumor metastasis through different mechanisms such as the induction of angiogenesis and ECM remodeling (178–181). In contrast, anti-tumor neutrophils can inhibit tumor growth through the direct killing of tumor cells via the production of ROS and NO or by trogocytosis of antibody-opsonized cancer cells (182, 183).

Besides their direct effect on tumor cells, TANs are engaged in dynamic and continuous interactions with a large variety of tumor infiltrating immune cells, affecting their phenotype and effector functions (14). In turn, these immune cells have a significant impact on neutrophil recruitment, phenotype and function (14, 17).

## Neutrophils and immune cells cross-talk in tumor

### Neutrophils and lymphoid cells

Neutrophils interact with a variety of lymphoid cells including CD4^+^ and CD8^+^ T cells, unconventional T cells, NK cells and B cells. In human samples, neutrophils have often been found co-localized with other lymphoid cells in the tumor bed or in the tumor-draining lymph nodes (139, 184–186).

**Figure 3:**
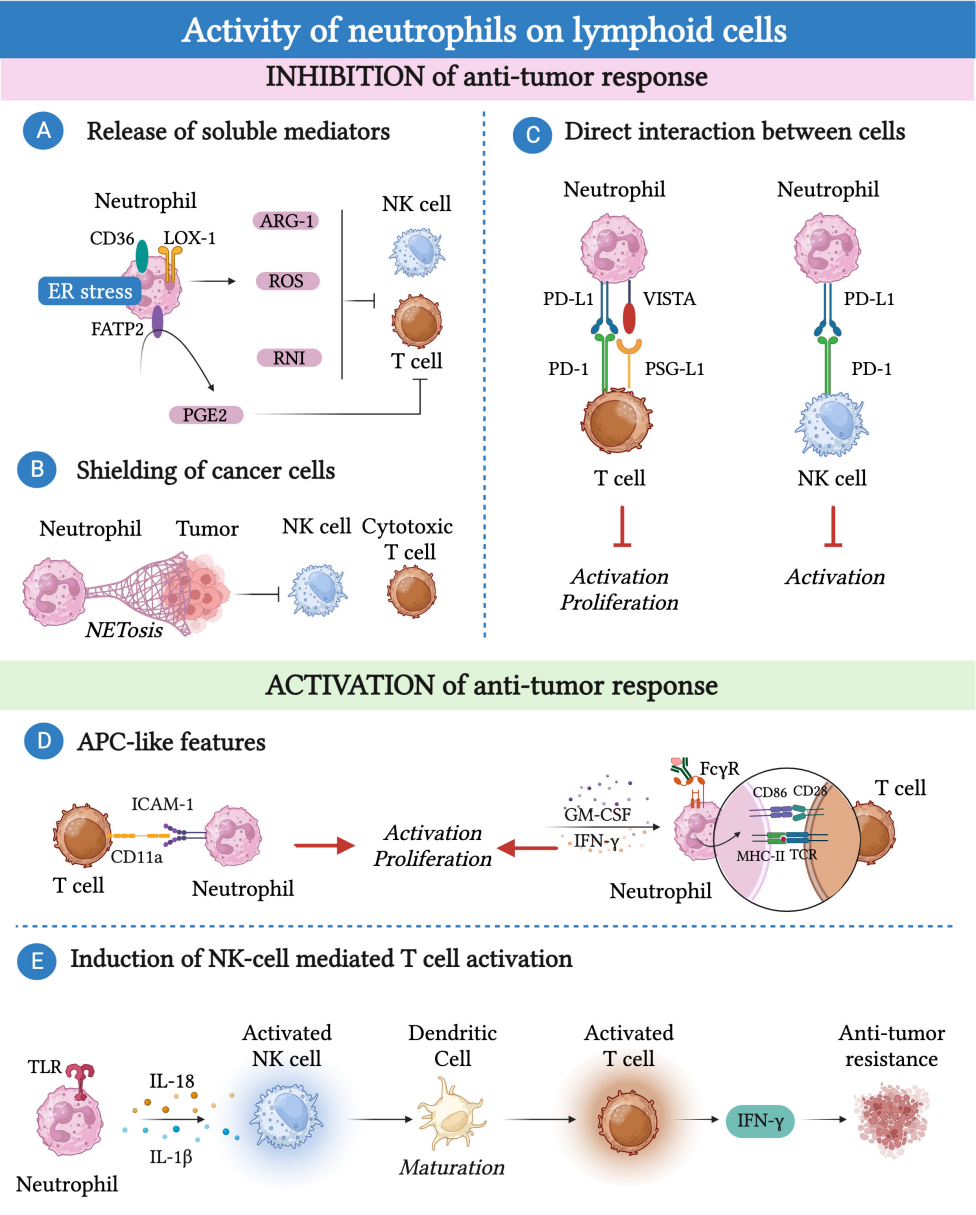
Neutrophils can promote or inhibit the anti-tumor response of lymphoid cells. **(A)** Immunosuppression can be mediated by neutrophils via the production of reactive oxygen species (ROS), reactive nitrogen intermediate (RNI) and arginase-1 (Arg-1) or through fatty acid transporter protein 2 (FATP2)-dependent production of prostaglandin E2 (PGE2). **(B)** Neutrophils can indirectly affect the cytotoxic activity of NK cells and CD8^+^ T cells by releasing neutrophil extracellular traps (NETs) that shield cancer cells. **(C)** Neutrophils express immune checkpoints such as programmed cell death 1 ligand 1 (PD-L1) or the V-domain immunoglobulin suppressor of T-cell activation (VISTA) that, by binding to their ligands PD-1 and P-selectin glycoprotein ligand-1 (PSG-L1), cause T cell and NK cell dysfunction. **(D)** Neutrophils can acquire antigen presenting cell-like (APC-like) features under the influence of GM-CSF or interferon (IFN)-γ or upon phagocytosis of antibody-antigen complexes via Fc gamma receptors (FcγRs). Similarly, neutrophils enhance TCR signaling in CD8^+^ T cells through the interaction between CD54/intercellular adhesion molecule 1 (ICAM-1) expressed on neutrophils and CD11a expressed on T cells. **(E)** Tool like receptor (TLR)-stimulated neutrophils can attract and activate NK cells which, in turn, trigger the maturation of dendritic cells resulting in T cell proliferation and IFN-γ production.

Neutrophils have the capacity to either inhibit or activate the effector functions of these lymphoid cells (**Figure 3**) (15).

Several lines of evidence have suggested that human and mouse neutrophil-derived soluble mediators, such as Arg-1, ROS, reactive nitrogen intermediates (RNI) and prostaglandin E2 (PGE2), played a key role in suppressing the effector functions of T cells and NK cells (17, 165, 187–193). In different mouse tumor models, TANs respond to TGF-β by producing significant amounts of Arg-1, leading to a reduction in the availability of L-arginine (165). Given the fundamental role of L-arginine in T cell metabolism, its deprivation results in T cell dysfunction (165). Accordingly, a population of neutrophils expressing Arg-1 has been found in renal cell carcinoma and NSLC patients with a frequency that negatively correlates with the frequency of CD8+ cytotoxic T lymphocytes (191, 192).

Changes in neutrophil metabolism may be linked to their pro-tumor or anti-tumor activities. In a transplantable mouse model of breast cancer with limited glucose supply, c-Kit^+^ immature neutrophils exhibited increased mitochondrial fatty acid oxidation, resulting in higher production of ROS and inhibition of the T cell response (187). The production of RNI, through iNOS–dependent NO production, was found to hinder T cell activation in mammary tumor-bearing *K14Cre; Cdh1^F/F^; Trp53^F/F^* (KEP) mice (189). Neutrophils found within the TME can exhibit endoplasmic reticulum (ER) stress and altered lipid metabolism (188, 194). This phenomenon has been associated with the expression of proteins involved in lipid trafficking and metabolism, such as CD36, lectin-like oxidized low-density lipoprotein receptor-1 (LOX-1), and fatty acid transport protein 2 (FATP2), and with an immunosuppressive phenotype of neutrophils (195). For instance, ER-stressed neutrophils produce higher amounts of ROS and Arg-1, which inhibit T cell proliferation and cytokine production (195, 196). Remarkably, FATP2 expression on neutrophils induced the production of PGE2, which has potent immunosuppressive activity on NK cells and CD8^+^ T cells (188, 197).

Neutrophils can also indirectly affect NK cell and CD8^+^ T cell cytotoxic activity through the release of neutrophil extracellular traps (NETs) that shield cancer cells (198). In this context, CXCL chemokines produced by tumor cells induce NETosis in neutrophils, which can coat and protect cancer cells from the cytotoxic activity of NK cells and CD8^+^ T cells. Interestingly, blockade of protein arginine deiminase 4 (PAD-4), which is essential for the formation of NETs, increased the activity of anti-PD-1 and anti-Cytotoxic T-Lymphocyte Antigen 4 (CTLA-4) immunotherapy in a transplantable mouse model of breast cancer (198).

The interactions between neutrophils and lymphocytes extend beyond the release of soluble mediators, as neutrophils themselves express immune checkpoints such as PD-L1 or VISTA. These molecules can interact with their ligands expressed on T cells and NK cells, leading to their dysfunction (199–204). Neutrophils expressing PD-L1 or VISTA have been found in various types of human and murine cancer, including hepatocellular carcinoma, melanoma, and gastric cancer (199–203).

As mentioned earlier, neutrophils can also interact with lymphoid cells to activate their anti-tumor activity. For instance, in patients with NSLC, neutrophils with antigen-presenting cell (APC)-like features were found and shown to be capable to activate CD4^+^ and CD8^+^ T cells (184). Neutrophils can acquire these APC-like features in response to TME-derived GM-CSF and IFN-γ, which induce the expression of MHC-II and CD86 in neutrophils (184). The interaction between these TANs isolated from lung cancer tissue and activated T cells led to increased expression of the costimulatory molecules CD54, CD86, OX40L, and 4-1BBL on the neutrophil surface, which further enhanced T cell proliferation, creating a positive feedback loop (205). Similar findings were observed in colorectal cancer patients, where neutrophils enhanced TCR signaling in CD8^+^ T cells, in a cell-to-cell contact dependent manner through the interaction between CD54/intercellular adhesion molecule 1 (ICAM-1) expressed on neutrophils and CD11a expressed on T cells (139). Further investigations have demonstrated that the phagocytosis of antibody-antigen complexes via Fc gamma receptors (FcγRs) renders murine and human neutrophils more potent APC-like cells (206).

In addition to T cells, neutrophils can induce NK cell activation through various mechanisms (207, 208). For example, cytokine-stimulated NK cells and neutrophils exchange contact-dependent activation signals involving CD18, ICAM-1 and ICAM-3 (207).

Stimulated neutrophils can attract and activate NK cells trough release of soluble mediators, including IL-1β and IL-18 (208). In turn, activated NK cells can trigger the maturation of dendritic cells, resulting in T cell proliferation and IFN-γ production, suggesting an additional mechanism trough which neutrophils can indirectly control the T cell anti-tumor immune response (208).

In addition to conventional T cells, neutrophils can influence the polarization and activation state of a subset of unconventional T cells, leading to their secretion of IFN-γ. This mechanism required a tripartite interaction between neutrophils, macrophages and unconventional T cells (see below) (143).

The reasons for these dichotomous functions of neutrophils on lymphoid cells are not fully elucidated. Interestingly, a recent study conducted in head and neck cancer (HNC) patients described this neutrophil dual role in tumor-draining lymph nodes, where neutrophils can interact with T cells in a stage-dependent manner (185). In metastasis-free patients, neutrophils transmigrate to lymph-nodes, acquiring APC-like features and promoting T cell anti-tumor activity (185). In contrast, at a later stage, neutrophils acquire PD-L1 expression and suppress T cell activation (185).

**Figure 4:**
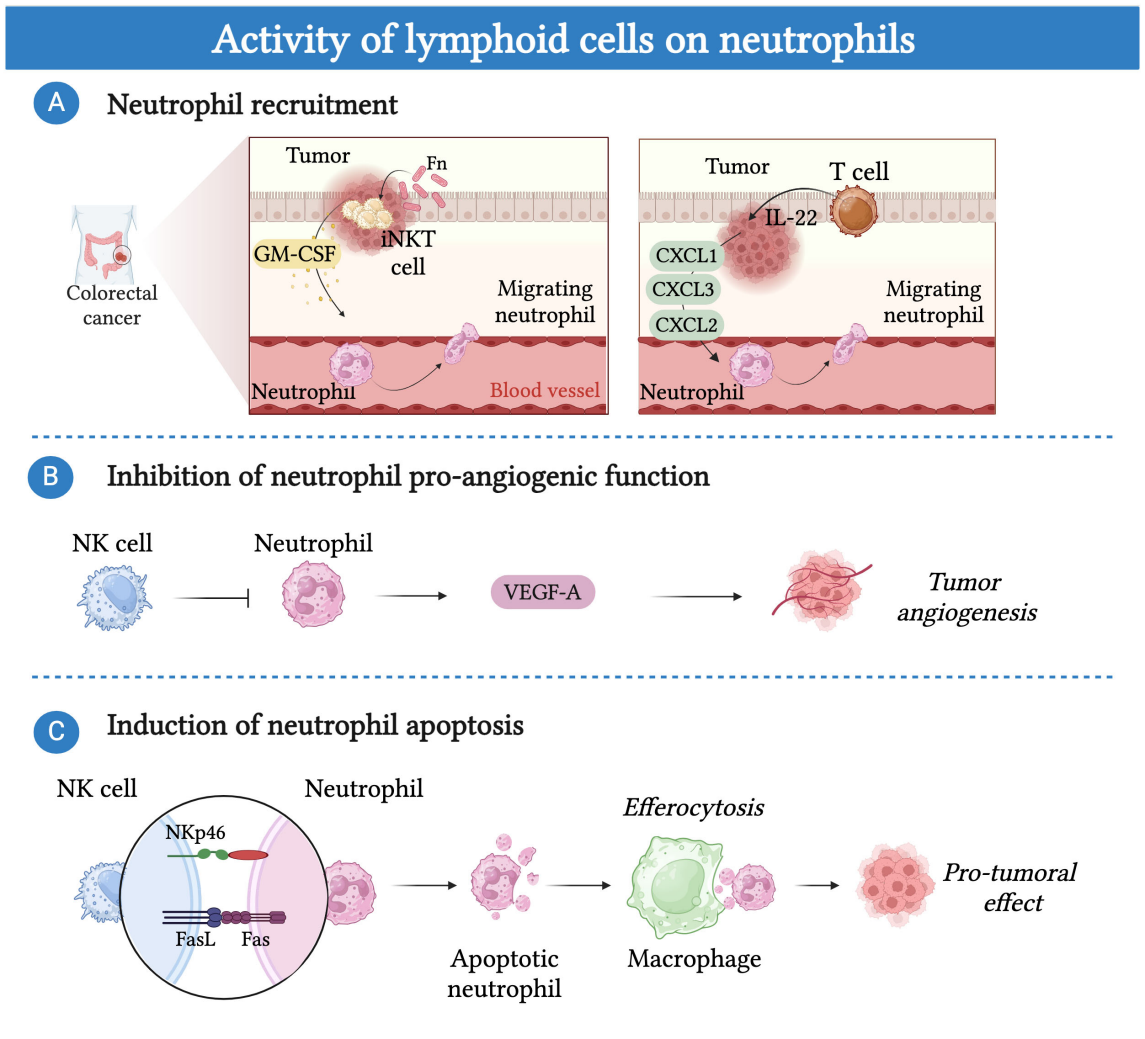
Lymphoid cells can modulate the activity of neutrophils. **(A)** Lymphoid cells can regulate the recruitment of neutrophils into the tumor bed. In CRC patients, tumor-associated pathobiont *Fusobacterium nucleatum* (Fn) induces the production of IL-17 and GM-CSF in invariant natural killer T cells (iNKT) resulting in increased neutrophil migration into the tumor bed. Additionally, IL-22 producing T cells induce the recruitment of neutrophils, by triggering the production of neutrophil-recruiting chemokines (i.e. CXCL1, CXCL2, CXCL3) by colorectal cancer cells. **(B)** NK cells control pro-tumor angiogenic function of neutrophils by blocking their secretion of vascular endothelial growth factor (VEGF). **(C)** NK-cells induce neutrophil apoptosis in a NKp46 and FAS-dependent manner. Efferocytosis of apoptotic neutrophils by macrophages promote a shift toward an M2-like pro-tumor phenotype.

Conversely, neutrophils can be influenced by lymphoid cells, which can modulate their recruitment and phenotype to the tumor bed under different conditions (**Figure 4**) (186, 189, 209, 210). For instance, in a mouse model of breast cancer in KEP mice, γδ T cells in response to IL-1β showed increased production of IL-17 which induced a G-CSF dependent accumulation of neutrophils with an immunosuppressive phenotype in the peripheral blood and metastatic lung (189). In CRC patients, IL-22 producing T cells induced the recruitment of neutrophils, by triggering the production of neutrophil-recruiting chemokines (i.e. CXCL1, CXCL2, CXCL3) by colorectal cancer cells. Importantly, the expression of IL-22 was found associated with the presence of neutrophils and T cells and a favorable prognosis (186).

Recently, in colorectal cancer patients, iNKT cells were found to increase the recruitment of neutrophils with immunosuppressive activity (209). The mechanism was related to the presence of the tumor-associated pathobiont *Fusobacterium nucleatum*, which induced the production of IL-17 and GM-CSF in iNKT. Importantly, the presence of iNKT cells and neutrophils correlated with a worse prognosis, suggesting that targeting this crosstalk could improve patient survival (209). On the other hand, NK cells have been involved in the control of neutrophil pro-tumor activity through an IFN-γ-dependent mechanism in mice (210). In a mouse model of transplantable sarcoma, the absence of NK cells induced neutrophils to acquire a pro-tumor phenotype characterized by the expression of VEGF-A (210) Interestingly, tumor-reprogrammed neutrophils that localize in a unique hypoxic and glycolytic niche exert a potent tumor-supporting, pro-angiogenic function through their high expression of VEGF-A (171). In contrast, NK cells have been suggested to induce neutrophil apoptosis via a NKp46 and FAS–dependent mechanism (211, 212). Remarkably, efferocytosis of apoptotic neutrophils by macrophages has been well documented to promote their shift toward an M2-like pro-tumor phenotype (213).

Neutrophils have been shown to interact also with B cells. Specifically, splenic neutrophils have been described to play a B-cell helper function, promoting the immunoglobulin class switching and the production of antibodies by activated B cells through a mechanism involving BAFF, APRIL and IL-21 (214). However, the involvement of this interaction in cancer has not been investigated.

### Neutrophils and myeloid cells

**Figure 5:**
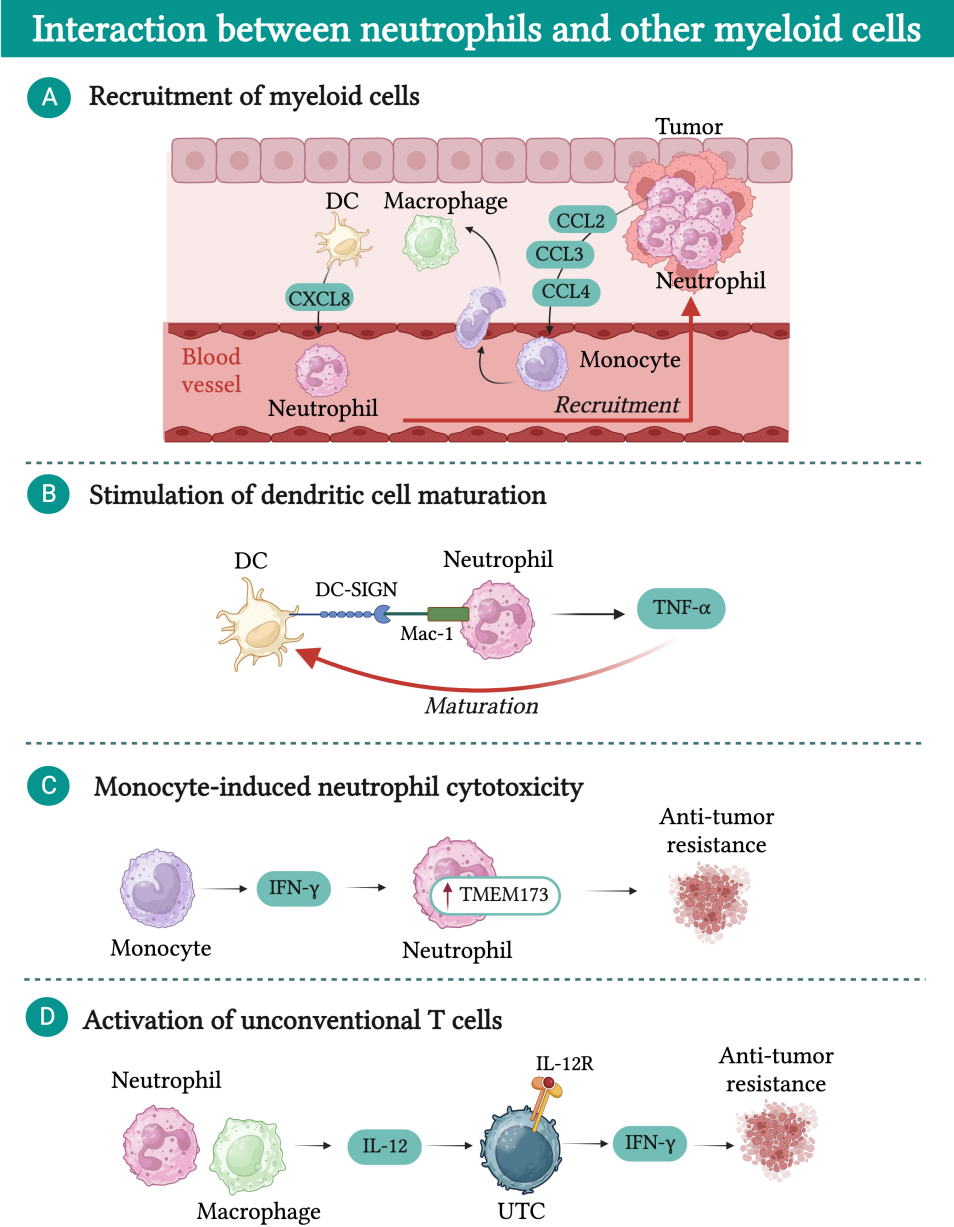
Interactions between neutrophils and other myeloid cells in cancer. **(A)** Macrophages and dendritic cells (DCs) are attracted into the tumor bed by chemokines released by neutrophils (e.g. CCL2, CCL3, and CCL4). In turn, macrophages and DCs produce chemokines (e.g. CXCL8) which further fuel neutrophil recruitment. **(B)** DCs physically interact with neutrophils via dendritic cell-specific intercellular adhesion molecule-3-grabbing non-integrin (DC-SIGN), resulting in increased release of tumor necrosis factor α (TNF-α). In turn, TNF-α promotes DC maturation. **(C)** IFN-γ produced by monocytes induces upregulation of transmembrane protein 173 (TMEM173) expression in neutrophils, unleashing their cytotoxic activity. **(D)** In a mouse model of sarcoma, neutrophil-macrophage interaction results in increased production of IL-12 by macrophages, which induces the expression of interferon γ (IFN-γ) in a subset of unconventional αβ T cells (UTCαβ) and favors their anti-tumor activity.

As mentioned above for macrophages, while a significant amount of research has focused on the interaction between neutrophils and lymphoid cells, a limited number of studies have explored the cross-talk between neutrophils and other myeloid cells in the TME (**Figure 5**) (215).

Neutrophils play a role in promoting the recruitment of other myeloid cells into the tumor bed through the release of chemokines such as CCL2, CCL3, and CCL4, which attract monocytes and dendritic cells (216). Once in the TME, monocytes and dendritic cells produce CXCL8, which favors the recruitment of additional neutrophils, creating a feedback loop that fosters the accumulation of inflammatory cells within the TME (217). Moreover, dendritic cells have been shown to physically interact with neutrophils via dendritic cell-specific intercellular adhesion molecule-3-grabbing non-integrin (DC-SIGN), leading to increased release of TNF-α by neutrophils (217). This interaction enhances the maturation of dendritic cells, thereby improving their capacity to effectively prime T cells and activate their anti-tumor response (217).

Another intriguing hypothesis is that neutrophils could amplify the source of antigens that dendritic cells process and present to T cells (218). Neutrophil-mediated trogoptosis of cancer cells may lead to an increased release of antigens and damage-associated molecular patterns (DAMPs) available for dendritic cells (219–222). This mechanism has been proposed as a potential therapeutic target and strategy to improve dendritic cell-based anti-cancer vaccines.

In a mouse model of breast cancer, it has been shown that neutrophil cytotoxic activity can be modulated by monocytes (223). Breast cancer cells with low spontaneous metastatic potential secrete high levels of CCL2, leading to the recruitment of IFN-γ-producing monocytes. Subsequently, neutrophils upregulate the expression of the transmembrane protein 173 (TMEM173, also known as stimulator of interferon response CGAMP interactor 1 (STING)), which then unleashes their cytotoxic activity (223).

Additionally, neutrophils were found to play a crucial role in potentiating the release of IL-12 by macrophages in the context of 3-methylcholanthrene (3-MCA)-induced sarcomagenesis (143). In turn, IL-12 can activate a subset of unconventional T cells (UTC) that express high levels of IL-12R, resulting in their production of IFN-γ and tumor control (143). To further underscore the importance of the interaction between neutrophils and other myeloid cells, two recent reports investigating the limited success of CSF-1R treatment in preclinical models of cancer revealed that upon TAM depletion, neutrophils acquired a highly immunosuppressive phenotype, counteracting the beneficial effect of macrophage depletion (224, 225).

## Conclusion and perspectives

The TME represents a complex ecological niche composed of tumor cells, stromal cells and immune cells constantly engaged in mutual interactions that influence tumor development and progression. TAMs and TANs are crucial components of this niche and affect tumor progression by directly influencing tumor cell proliferation and by shaping the anti-tumor or pro-tumor response of other immune cells. In turn, the phenotypes and activities of TAMs and TANs are continuously regulated by other immune cells present in the TME.

Targeting the immune system represents a therapeutic strategy against cancer. However, a significant percentage of patients do not respond to current available treatments, underlining the need for new therapeutic approaches. As we uncover the complexity of the interactions between macrophages and neutrophils and other immune cells, it becomes evident that targeting these interactions may hold promise for developing novel and effective immunotherapeutic approaches to fight cancer.

## Author contributions

ID: Writing – original draft. SC: Writing – original draft. AR: Writing – original draft. GG: Writing – original draft. PM: Writing – original draft. SJ: Writing – review & editing.
